# Factors associated with retention in HIV care at Sediba Hope Medical Centre

**DOI:** 10.4102/sajhivmed.v16i1.347

**Published:** 2015-07-01

**Authors:** Nishana Ramdas, Johanna C. Meyer, David Cameron

**Affiliations:** 1 Department of Pharmacy, Sefako Makgatho Health Sciences University, South Africa; 2 Foundation for Professional Development, Pretoria, South Africa; 3 Department of Family Medicine, University of Pretoria, South Africa

## Abstract

**Background:**

Lost to follow-up (LTFU) is a major challenge that hinders the success of antiretroviral treatment (ART).

**Objective:**

To identify factors conducted to a low LTFU rate.

**Methods:**

We conducted a two-part descriptive and quantitative study. Part 1 comprised interviews with clinic staff to determine their perspectives on LTFU and to establish the clinic’s follow-up procedures for patients on ART. Part 2 of the study was a retrospective review of clinic and patient records. LTFU patients were identified and those with contact details were contacted for telephonic interview to determine if they were still on ART and/or their reasons for becoming LTFU.

**Results:**

A low LTFU rate (7.9%; *N* = 683) was identified. Work-related stress, and lack of transport and funds were reported reasons for LTFU. Monthly visits, non-adherent defaulters and LTFU patients were tracked by an electronic system (SOZO). Factors contributing to high rates of retention in care were: location of the clinic in the inner city, thus in close proximity to patients’ homes or work; clinic operating on Saturdays, which was convenient for patients who could not attend during the week; an appointment/booking system that was in place and strictly adhered to; a reminder SMS being sent out the day before an appointment; individual counselling sessions at each visit and referrals where necessary; and a stable staff complement and support group at the clinic.

**Conclusion:**

Achieving a low LTFU rate is possible by having a patient-centred approach and monitoring systems in place.

## Introduction

Adhering to antiretroviral treatment (ART) is a lifelong commitment that requires patients to diligently adhere to daily medication dosing schedules and make regular clinic visits for care.^1^ ART has improved the lives of many people living with the human immunodeficiency virus (HIV), but many challenges still exist before ART programmes might achieve total success in terms of patient outcomes.^1^ Two of the major challenges and concerns for ART programmes are retention in care and patients who are lost to follow-up (LTFU).^1^ Several studies have been conducted on these problems, investigating various ways to improve retention in HIV care and patient outcomes.^1,2,3,4^

‘Lost to follow-up’ refers to the disappearance of a patient from the programme, for no reported reason.^5^ Definitions of when a patient is classified as LTFU vary widely across studies and countries.^6^ In a pooled analysis of 111 facilities, a threshold of 180 days since the last clinic visit was recommended as a standard definition for LTFU.^7^

In sub-Saharan African countries, rates of LTFU vary extensively. According to a systematic review of patient retention following ART initiation, it was evident that 1 year after initiation approximately 25% of patients were no longer in care, with LTFU figures escalating to 40% after 2 years.^8^ Lower LTFU figures (3.3%; *N* = 2548) were reported from a retrospective cross-sectional study of a community-based ART cohort in Cape Town, South Africa, which used a computerised tracking tool to manage patients in care.^9^

Sediba Hope Medical Centre (SHMC) is a nongovernmental organisation (NGO) clinic, situated in the city centre of Tshwane in South Africa. It caters for patients working and living in and around the city of Tshwane. The centre was previously known as Fountain of Hope (FOH), which was a Foundation for Professional Development (FPD) clinic funded by the President's Emergency Plan for AIDS Relief (PEPFAR) and the United States Agency for International Development (USAID). The clinic's budget allowance provided for a patient database of only 500 patients. The main purpose of the FOH Clinic was to provide ART for HIV-positive patients living and working in the inner city. In 2011, FPD joined in a project with Participate Empower and Navigate (PEN), a non-profit, non-denominational, faith-based organisation, and subsequently the FOH Clinic became the SHMC. Since the name change, it has operated as an ARV site for the PEPFAR- and USAID-funded patients on ART and as a primary healthcare (PHC) private practice for patients using medical aid or paying privately. The move from the FOH Clinic to the SHMC had some implications for patients as they had to adapt to a new site, new processes, new staff and additional travelling distances for some patients. Patients living near the FOH Clinic had to walk an average of 1.4 km further to the SHMC, which represents approximately 17 minutes.

LTFU had not been evaluated at this site previously. In the present study, we aimed to quantify LTFU at the SHMC, investigate the factors that contributed to patients on ART becoming LTFU, and identify factors that could contribute to low LTFU rates and be implemented to improve retention in care.

## Methods

### Patient population and data collection

We conducted a two-part descriptive and quantitative study at SHMC between August and November 2013. The first part of the study included an individual interview based on a structured questionnaire with each of the nine staff members. We aimed to determine staff members’ perspectives on the reasons for LTFU and to establish the procedures used at the clinic to monitor patients on ART and to identify and trace those who were LTFU.

The second part of the study was a retrospective review of clinic and patient records for the previous 4 years (2010–2013). The review included ‘hard copy’ patient files and an electronic patient management system, known as SOZO, which was developed by FPD in partnership with Infocare and John Snow International (JSI) in 2007. All patients on ART, who had become LTFU with no obvious reason for default, were identified. A patient record sheet was used to record LTFU patients’ details from their files and the SOZO system. Patients identified as LTFU, with contact details in their records, were contacted telephonically for a structured telephonic interview to investigate reasons for LTFU.

### Data analysis

Data were analysed with SPSS V21.0 statistical software. The percentage of LTFU patients was calculated with a 95% confidence interval. Patient demographics and clinical, treatment and social data were summarised descriptively. Associations between variables and differences in means were identified with Fisher's exact test and independent samples *t*-test as appropriate. Statistical significance was set at *p* ≤ 0.05.

## Ethical approval

Ethical clearance for the study was granted by the Medunsa Research Ethics Committee of the University of Limpopo. Permission to conduct the study at the SHMC was provided by FPD and the manager of SHMC. Written consent was obtained from staff members of SHMC and verbal consent from responding patients, prior to their participation in the interviews.

## Results

SHMC is conveniently situated in the city centre; that is, close to home or work for most patients. The clinic operates on weekdays as well as Saturdays to accommodate patients who work or are unable to attend the clinic during the week. The previous FOH Clinic also hosted a support group with regular meetings every second Saturday, which were available to all patients from the time they started treatment.

### Patient flow process and tracking of lost to follow-up patients

**FIGURE 1:**
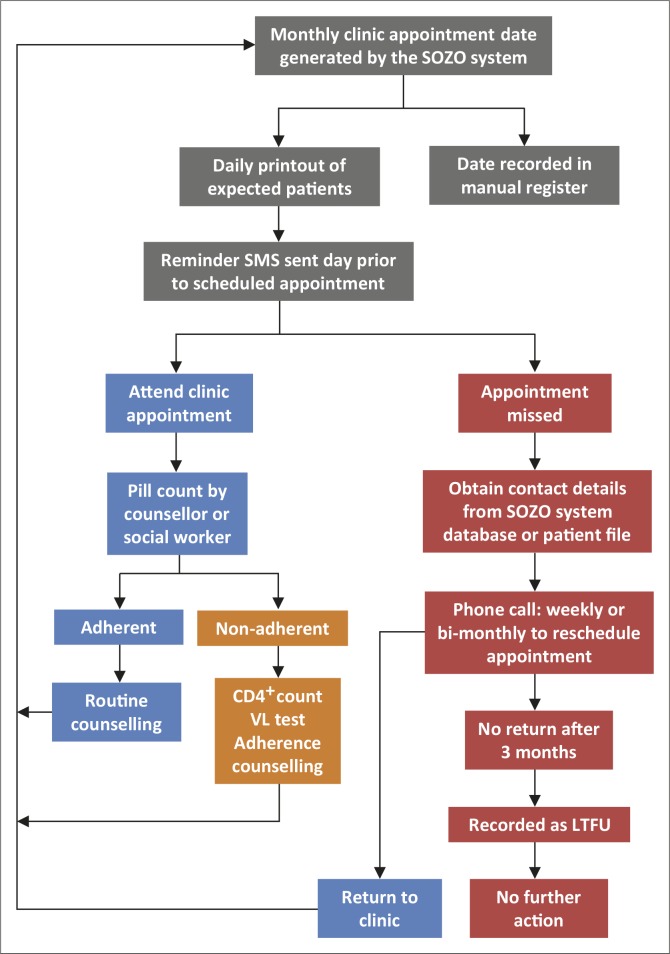
Flow process of monitoring and tracking patients on antiretroviral treatment at Sediba Hope Medical Centre.

Interviews with staff members revealed that the clinic followed a structured patient flow process, according to which patients were seen by appointment only and according to bookings done on the SOZO system. According to the staff interviewed, patient waiting time was kept to a minimum, as appointments were made according to a time schedule. The patient flow process and the tracking of LTFU patients at SHMC are illustrated in Figure 1.

Patients at SHMC are sent a reminder short message service (SMS) the day prior to their scheduled appointment. A booking list is printed daily to track patients as they attend. The service received is personalised and, should patients not attend their appointment, follow-up phone calls are made.

When a patient on ART is identified as LTFU, the social worker phones the patient weekly or at least bi-monthly for a period of 3 months, in an attempt to reschedule an appointment and get the patient back into care. All phone numbers on patient records are contacted in an attempt to trace them. After 3 months of no success in tracing a patient, no further action is taken.

The staff complement at the previous FOH Clinic was stable for a period of 4 years and, consequently, patients interacted with the same healthcare worker at each visit. For this reason, patients felt comfortable and built good relationships with the staff.

#### Extent of lost to follow-up

**FIGURE 2:**
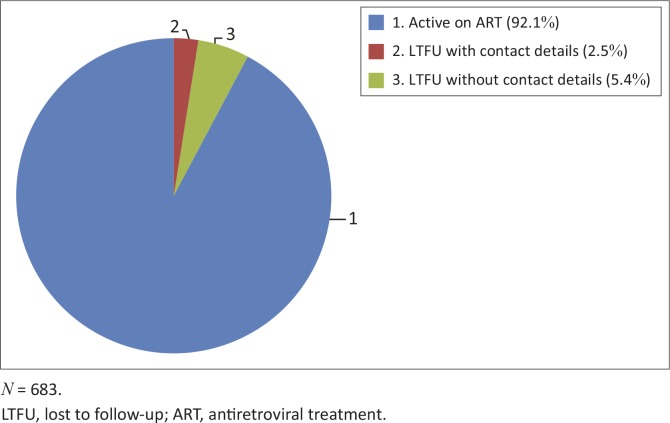
Overall proportion of patients lost to follow-up at Sediba Hope Medical Centre.

Of the total number of 683 patient records reviewed, 54 (7.9%; 95% confidence interval [CI], 6.1%–10.2%) patients were identified as LTFU (Figure 2). Only 17 of the 54 patients had contact details in their records and were contacted for a telephonic interview. Sociodemographic characteristics of the 54 patients who were LTFU are summarised in Table 1.

**TABLE 1:** Sociodemographic characteristics of lost to follow-up patients.

Sociodemographics	Characteristics	*n*	%
Gender	Male	26	48.1
	Female	28	51.9
Marital status	Single	28	51.9
	Married	21	38.9
	Widowed	1	1.9
	Divorced	1	1.9
Educational level	No education	2	7.1
	Primary education completed	1	3.6
	Secondary education not completed	9	32.1
	Secondary education completed	11	39.3
	Tertiary or vocational education	5	17.9
Employment status	Employed	23	42.6
	Self-employed	6	11.1
	Unemployed	20	37.0
	Student/scholar	2	3.7

*N* = 54.

#### Disclosure of HIV status

According to the patient records, most patients (90.7%; *N* = 54) had disclosed their HIV status. Women preferred to disclose to a relative (82.1%; *n* = 28) rather than a partner (28.6%; *n* = 28) whilst men disclosed mainly to relatives (46.1%; *n* = 26) and their partners (42.3%; *n* = 26). Three male patients did not disclose their status to anybody.

#### Travelling distance to the clinic

Travelling distance to the clinic was known for 16 of the 17 LTFU patients who were contacted for an interview. Nine of them (56.3%) lived less than 1 km away from the clinic, and so required minimal travelling; 3 (18.8%) lived between 1 km and 5 km from the clinic; and 4 (25.0%) lived more than 10 km from the clinic.

#### Clinical profile of lost to follow-up patients: CD4^+^ cell count and viral load

Table 2 shows the gender distribution of LTFU patients, for whom CD4^+^ cell count test results were available within 6 months of their final clinic visit, prior to becoming LTFU.

**TABLE 2:** Distribution of patients according to CD4^+^ cell count results, based on testing done within 6 months of the patient's final clinic visit prior to becoming lost to follow-up.

CD4^+^ count (cells/μL)	Male†	%*	Female‡	%*	Total§	%
< 100	2	5.7	2	5.7	4	11.4
100–350	15	42.9	5	14.3	20	57.2
> 350	1	2.9	10	28.6	11	31.4
Range	11–1382		69–656		11–1382	
Median	255.0		431.0		289.0	
IQR	173.25–299.0		253.0–471.5		186–438	
Mean ± s.d.	287.8 ± 287.6		371.9 ± 167.0		328.6 ± 237.3	
Mean difference (95% CI)	84.1 (−247.1–78.9)**					

†, *n* = 18; ‡, *n* = 17; §, *N* = 35.

*, *p* = 0.001, Fisher's exact test; **, *p* = 0.302, independent samples *t*-test.

The median CD4^+^ count for patients (*n* = 35) who had a test done within 6 months of their last clinic visit prior to becoming LTFU was 289.0 cells/μL (interquartile range [IQR] 186–438), with a CD4^+^ count below 350 cells/μL for the majority (68.6%) of them. Categorisation of CD4^+^ count results available within 6 months of the final clinic visit prior to becoming LTFU showed a statistically significant association with gender (*p* = 0.001; Fisher's exact test). Being female was associated with the probability of a CD4^+^ count > 350 cells/μL, and being male was associated with a probability of a lower CD4^+^ count (100 cells/μL – 350 cells/μL) at the time of being LTFU.

A detectable viral load (VL) within 6 months of their final visit to the clinic was evident in 44.1% (*n* = 35) of patients, with more of the men (58.8%; *n* = 17) than the women (29.4%; *n* = 17) having a detectable VL. The association between gender and detectable VL was, however, not significant (*p* = 0.472; Fisher's exact test).

#### Antiretroviral treatment and adherence

At the time of their final visit, just more than half of LTFU patients (57.4%; *n* = 54) were on the current first-line regimen, comprising tenofovir (TDF), lamivudine (3TC) and efavirenz (EFV). Thirteen per cent of the LTFU patients were still on the previous Regimen 1A (stavudine [d4T], 3TC, EFV) and 11.1% on the previous Regimen 1B (d4T, 3TC, nevirapine [NVP]). Only 13% of LTFU patients were on the second-line regimen (TDF, 3TC or emtricitabine [FTC] and lopinavir/ritonavir [LPV/r]).

Adherence to ART at the SHMC was monitored at each visit by means of pill counts, conducted by the social worker or counsellor and recorded as descriptive notes in the patient files and on the SOZO system. Table 3 shows the adherence patterns for patient visits over the period of 3 months prior to becoming LTFU.

**TABLE 3:** Adherence patterns over the period of 3 months prior to becoming lost to follow-up.

Adherence level	Number (%) of patient visits							
	Third-last month†	%	Second-last month‡	%	Month prior to LTFU§	%	Total	%
Excellent adherence	10	23.9	4	9.1	2	4.3	**16**	**12.0**
Adherent	27	64.3	34	77.3	30	63.8	**91**	**68.4**
Non-adherent	5	11.9	6	13.6	15	31.9	**26**	**19.6**

*N* = 133; †, *n* = 42; ‡, *n* = 44; §, *n* = 47.

#### Reasons for lost to follow-up

Staff members’ perceptions of the reasons for LTFU are summarised in Table 4.

**TABLE 4:** Reported reasons for lost to follow-up, according to staff perceptions.

Reasons for lost to follow-up	Number
Work-related stress (e.g. cannot take leave to attend clinic)	7
Lack of transport	5
Only one month's supply of ART (opposed to 3 months’ supply issued previously)	5
Strenuous/tedious to attend clinic every month	2
Stigma/shame	1
Foreigners going back home	1
Patients moving to other provinces	1
Domestic abuse	1

*N* = 9.

Some participants provided more than one reason.

Based on the contact details available in the files of patients identified as LTFU, only 17 patients could be contacted successfully for a telephonic interview. One patient was identified to have demised while on ART. Another patient had relocated to Europe, and it could not be determined whether he was still on ART or not.

Nine patients were confirmed to be still active on ART at other sites, of whom one patient was actually LTFU at SHMC before initiation on ART. This patient subsequently commenced ART at another site, as he moved from one city to another. Three patients indicated that they were using delivery services to obtain their ARVs. Explanations from the remaining five patients who continued treatment at a different ART facility were related to proximity and travelling time. Two of these patients reported that the alternative facility was closer to home, and three reported it as being closer to their workplace.

Only six of the patients interviewed reported that they had discontinued ART altogether. All six patients provided transport costs as a reason for discontinuing treatment; 2 of these patients also mentioned the side-effects of ARVs, whilst one patient was using traditional or herbal medication instead of ARVs.

## Discussion

The LTFU rate at the SHMC (7.9%; *N* = 683) was evidently much lower in comparison to most sub-Saharan countries.^1,9^ This reasonably low LTFU rate could be attributed to various processes at the centre, one being the follow-up of patients from an early stage after a missed appointment at the clinic. A previous study confirmed that early active follow-up of patients can improve retention on treatment and programme outcomes.^9^

The use of an electronic patient management system (SOZO) facilitated patient follow-up and engagement, thus improving the efficiency of the system immensely. Appointments were booked electronically according to a time schedule which, according to the staff, minimised patient waiting time. A large ART programme in Malawi also considered time-specific appointments for each patient as an option to reduce waiting times.^2^ In addition, the SOZO system identified patients due for appointment, and a reminder SMS was sent to them the day beforehand. Similarly, low LTFU rates (3.3%) were identified elsewhere in South Africa where a computerised pharmacy tracking system (iDART) was used to trace patients who failed to collect their medication.^9^

Most (90.7%) of the LTFU patients disclosed their HIV status, which was a positive finding and attributed to the fact that disclosure is encouraged during counselling. Disclosure of HIV status to one's spouse is known to be associated with good adherence.^10^

Women in our study had a higher CD4^+^ count at the time of being LTFU. This could be expected as previous studies from sub-Saharan Africa have shown that women usually have a higher CD4^+^ count at ART initiation and a better median CD4^+^ count increase from baseline across all time periods after ART initiation, than men.^11^

It is evident that an elevated VL may be a factor to consider for LTFU, which is supported by the findings from other studies which demonstrated that unsuppressed viral loads at any time point in treatment are predictive of loss.^12^

Good adherence rates were supported by consistent pill counts and counselling sessions, which happened at each clinic visit. Having a stable staff complement at the clinic meant that patients saw the same counsellor or social worker at every visit, which facilitated good relationships between staff and patients. The staff perceived the regular support group meetings as a contributing factor to adherence. Support groups are known to encourage adherence and improve retention in care.^13^

From our findings, it was apparent that work-related stress, lack of transportation, and lack of funds for travel and food were reported as contributing factors to LTFU at SHMC. Travelling distance was the main reported reason why patients changed facilities and opted for a clinic closer to home or to work, or preferred the convenience of ART delivery services to their home or work. High transport costs and patients having to travel long distances to get to ARV clinics were identified as problems in a study conducted at Themba Lethu Clinic, Helen Joseph Hospital, in Johannesburg, South Africa.^3^ Lack of transport and employment obstacles as reasons for LTFU are supported by a number of other studies conducted in South Africa and Mozambique.^3^^,14,^^15^

Lack of availability of contact details for all patients at SHMC made follow-up and tracing of LTFU patients difficult in our study. The majority of LTFU patients had incorrect information or no contact information at all. It is apparent that ART programmes should invest in obtaining accurate, complete and up-to-date contact details for patients to aid tracing. Availability of more updated contact information for all patients at SHMC may have resulted in an even lower number of LTFUs altogether.

### Limitations

Our study was conducted at only one facility and the results can therefore not be generalised to other ART facilities in South Africa. Incomplete and incorrect patient records (manual and SOZO system) made it difficult to trace all LTFU patients and therefore negatively affected the number of LTFU patients interviewed. A limitation of the study itself was that the review did not include patients who remained in care. Consequently, comparisons between LTFU patients and those who remained in care were not possible. Furthermore, as a result of incomplete clinic records, information about the proportion of patients who did not become LTFU as a result of successful contact by social workers could not be determined.

## Conclusion

From our study, it is evident that low LTFU rates and measures to prevent LTFU are possible. The flow through the clinic was efficient and patients in general were pleased with the services rendered at SHMC. Most patients had built good relationships with the staff, which made them feel comfortable and cared for. The complete functioning of SHMC took the form of a patient-centred approach and was much more than only having a computer system in place.

SHMC can be an example to similar ART sites with high LTFU rates. The SMS reminder service and tracking system may benefit other ART sites. Seeing patients on an appointment only basis proved to be beneficial. However, it might be difficult in facilities with larger numbers of patients.
